# Cases of Aneurism from Bleeding

**Published:** 1827-02

**Authors:** B. C. Brodie

**Affiliations:** St. George's Hospital


### ANEURISM.

### *Cases of Aneurism from Bleeding, which were treated at* St. George's Hospital, *under the care of* B. C. Brodie, Esq. &c. &c.

Case I.—J— R———, ætatis forty-two, on the evening of the 30th of August, 1826, was bled in the arm. Unfortunately, the lancet penetrated the coats of the brachial artery. A copious hemorrhage followed, which was with some difficulty restrained by the application of compresses and a bandage. On the 1st of September, he was admitted into St. George's Hospital. At this time the arm was very much swollen, in consequence of the extravasation of blood into the cellular texture, and there was an undefined pulsating tumor on the fore part of the elbow-joint.

Soon after the man's admission into the hospital, and thirty-six hours after the occurrence of the accident, Mr. Brodie performed the following operation:

In consequence of the extensive extravasation of blood which had taken place among the muscles of the arm, he did not attempt to secure the artery at a distance from the puncture. An incision was made on the inner edge of the biceps flexor cubiti, so as to expose the artery at the part at which the lancet had entered. A ligature was then applied round the vessel, immediately above the puncture. On the tourniquet being loosened, it was found that this ligature was not sufficient to restrain the hemorrhage. A second ligature was therefore applied below the puncture.

This operation was not unattended with difficulty, owing to the large quantity of coagulated blood which lay over and around the wounded vessel. The wound was dressed to the bottom with lint, as there seemed to be no probability of union taking place by the first intention. Inflammation of the arm, with some degree of febrile excitement of the system, followed the operation.

On the 5th of September, the lint was removed from the wound. A considerable quantity of matter escaped, which gave the patient immediate relief.

On the 6th of September, there was but little pain; the tension of the limb had in a great degree subsided. The pulse could not be perceived at the wrist.

Sept. 11th.—One ligature was separated; and the other came away two days afterwards.

From this time a progressive amendment took place, until the 26th of September, when there was a slight erysipelatous inflammation of the arm, which however subsided in the course of two or three days. On the 30th of September, the patient left the hospital, the wound being nearly healed.

In this case it was observed that the brachial artery was wounded immediately above its division into the arteries of the fore-arm, and the puncture was of a considerable size. This last circumstance accounts for the unusual degree of extravasation which followed the accident. The operator would have cut down on the artery in the arm, if he had not found that this operation was likely to have been attended with more difficulty than that of exposing the vessel on the fore part of the elbow-joint. It is probable, however, that, even if the operation had been done in the first of these ways, it would have been found to be necessary to secure the artery below the puncture also, as the ligature applied close to and immediately above the puncture was insufficient to restrain the hemorrhage.

The following case, which occurred in St. George's Hospital a few years ago, is here related, as it forms another instance showing that the application of a single ligature above the wound of the artery is not always equal to the cure of this kind of aneurism.* There are also some other points in the case which are well worthy the notice of the pathologist.

Case II.—H. Chambers, a young man, had been bled in the arm while residing in the West Indies, in the year 1820. The fore-arm was observed to be black afterwards, and in the course of two or three days a small pulsating tumor was discovered on the fore part of the elbow-joint. By degrees the tumor attained the size of a large walnut. In April 1820, a surgeon applied a ligature round the brachial artery, three or four inches above the tumor. After this, according to the patient's account, the pulsation ceased, and the tumor became diminished in size; but the operation was followed by severe pain, and at the same time by a sense of numbness of the hand. The pain subsided in the course of a few weeks, but the numbness remained. At the end of two or three months, it was discovered that the pulsating tumor had returned. The tumor increased in size, and the patient came to England, and was admitted into St. George's Hospital in the beginning of September 1820.

At this time there was a pulsating tumor on the fore part of the elbow, of the size of a pigeon's egg. On making a light pressure

* Mr. Brodie observed, that in another case in which he had tied the brachial artery in the middle of the arm, on account of an aneurism from bleeding in the usual situation, although the immediate effect of the ligature was to stop the pulsation of the aneurism, the pulsation returned on the following day, and continued (more feeble, however, than before the operation,) for nearly a month. After that period it gradually ceased, and the tumor disappeared.

on it with the ends of the fingers, besides the pulsation, a peculiar thrilling sensation was perceptible, different from that which is perceived in common aneurism, and corresponding to that which is described as being produced by a varicose aneurism.

On the 7th of December, Mr. Brodie performed the following operation:—He made an incision on the brachial artery, one inch and a half above the aneurism, and tied that vessel with a single silk thread. At first it was supposed that the pulsation of the aneurism had ceased: in a few minutes, however, the pulsation returned, though not quite so strong as before the application of the ligature. A tourniquet was then applied in the arm, and an incision was made into the aneurismal sac. Some coagulum which lay in the sac having been removed, the inner surface of the sac was seen lined by a thin, white, polished membrane. On the tourniquet being loosened, arterial blood flowed through a large orifice which was manifestly that of the brachial artery. A second ligature was therefore applied to this vessel, immediately above the aneurism. There were still two other orifices communicating with the sac. One of these was manifestly that belonging to the lower portion of the brachial artery, through which also arterial blood flowed in a considerable stream, on the tourniquet being loosened. Another ligature was therefore applied to the brachial artery, immediately below the aneurism. Still, on loosening the tourniquet, blood flowed through the third orifice; and it was observed that there was here a double current, composed partly of venous, partly of arterial blood. It was evident, therefore, that this orifice communicated with both an artery and vein; and, the blunt end of a probe having been introduced into it, so as to mark its situation, a fourth ligature was applied close to the sac, so as to intercept at once the communication with both of these vessels.

The edges of the wound were brought together by means of straps of adhesive plaster. The ligatures separated at the end of ten days, and the wound healed without any evident suppuration of the inner surface of the aneurismal sac. The patient quitted the hospital cured in about a month after the operation.

From what was observed in this case, we must conclude that the aneurism communicated not only with the wound of the artery, but with that of the vein also, so that it might be considered as a complication of common and varicose aneurism. This complication has been noticed by Dr. Hunter, and appears to be not uncommon.

An example of it was met with in a patient who died in St. George's Hospital, in the year 1818, under the care of the late Mr. Griffiths, two or three months after he had been wounded by an iron spike, which entered the groin, and penetrated under Poupart's ligament. There was a puncture of the external iliac vein, and another of the external iliac artery, and a large aneurismal sac which communicated with both of these vessels.

